# Effect of Immunization Against Inhibin on Camel Testicular Morphometry, Echotexture Analysis, Semen Quality, and Vascularization in Relation to Hormonal Aspect

**DOI:** 10.3390/vetsci12090896

**Published:** 2025-09-15

**Authors:** Elshymaa A. Abdelnaby, Hossam R. El-Sherbiny, Abdulrhman K. Alhaider, Mohamed Marzok, Ibrahim A. Emam

**Affiliations:** 1 Department of Clinical Sciences, College of Veterinary Medicine, King Faisal University, P.O. Box 400, Al-Ahsa 31982, Saudi Arabia; alhadier@kfu.edu.sa (A.K.A.); mmarzok@kfu.edu.sa (M.M.); 2 Theriogenology Department, Faculty of Veterinary Medicine, Cairo University, Giza 12211, Egypt; hossamelsherbiny7353575@cu.edu.eg; 3 Department of Surgery, Anesthesiology and Radiology, Faculty of Veterinary Medicine, Cairo University, Giza 12211, Egypt; dr.ibrahimabdallah2018@cu.edu.eg

**Keywords:** camel, hemodynamics, testicular artery, immunization, inhibin

## Abstract

This study aims to demonstrate whether immunization against inhibin could alters testicular echotexture (TE), hemodynamics (resistance and pulstalilty index [RI and PI], testicular blood flow volume [TBFV]), and semen picture. Senile male camels were immunized actively against inhibin alpha subunit (immunized males; n = 5; subcutaneous route; 1 mL) or non immunized (control males; n = 5). The injection was four times with four weeks intervals. Semen was collected by electroejaculator once per week. Blood sampling, ultrasonography, and hormonal assaying was performed once per week. The levels of follicle stimulating hormone (FSH), luteinizing hormone (LH), estradiol (E2), testosterone (T), and nitric oxide (NO) were calculated. Regarding control camels, the FSH levels were elevated (*p* < 0.01) in immunized males weeks 6–9 in August. Both E2 and NO were increased in the same group at weeks 7–11 with the highest level in week 10 for E2 (17.01 ± 0.11 pg/mL) in September and in week 9 for NO (44.66 ± 0.15 µmol/L). The Doppler indices RI and PI were declined (*p* < 0.01) in immunized camels in weeks 7–11, while the TBFV was elevated (*p* < 0.01). A marked elevation (*p* < 0.05) in testicular volume was noticed in the immunized group with a decline in the TE in week 9. The sperm cell concentration and viability were elevated (*p* < 0.01) in the immunized group. In conclusion, the active immunization against inhibin in senile camels shows a positive effect via improvement of testicular hemodynamics, flow volume, testicular volume, FSH, E2, NO, and sperm cell concentration with viability %, and alterations in both Doppler indices with the TE.

## Simple Summary

The testicular blood flow, echotexture, and Doppler indices were examined in senile male camels immunized actively against inhibin alpha subunit at four times with four weeks intervals. The FSH levels were elevated in immunized males from week 6 to 9 in August. Both E2 and NO were increased in the same group in weeks 7–11 with a highest level at weeks 9–10. The sperm cell concentration and viability were elevated (*p* < 0.01) in the immunized group. This study demonstrated that an active immunization against inhibin in senile camels shows a positive effect via improvement of testicular hemodynamics, flow volume, testicular volume, FSH, E2, NO, the sperm cell concentration with viability percentage, and alterations in both Doppler indices with echotexture.

## 1. Introduction

Aging could decline animal semen quality and reproductive hormones as many changes in pituitary gonadal axis were integrated in aging pathophysiology [1,2,3]. Since the seminiferous tubules make up to 80% of the testicular mass with a very low oxygen content, adequate blood supply is very critical to the testis’s ability, vascularity, and functionality [4,5]. According to previous research, ischemia damages to the testis and spermatogenesis deficiencies can result from reduced testicular blood flow because of defective energy metabolism in the testicular microcirculation [6,7]. Animal fertility benefits from a variety of therapies that increase testicular blood flow, which in turn enhances the testicular function [8,9,10]. From those different methods of testicular function assessment, the color Doppler could be perfectly used. Color and pulsed wave Doppler modes could be used as non-invasive tools to enhance the reproductive efficiency in animals [11]. The Doppler ultrasound was used to evaluate the reproductive organs functionality via accurate estimation of blood flow volume (BFV) and the vascularity index represented by resistance and pulstatilty indices (RI an PI) [12].

This could be achieved in many studies in female reproduction [13,14], and male reproductive ability accompanied with fertility via color coding intensity that related to flow direction [15,16]. Beside the importance of grey image analysis, the testicular echotexture represented by echogenicity (TE; NPVs) could be used in selecting some animals for breeding [17].

The follicle stimulating hormone (FSH) is an important hormone in spermatogenesis. As it could alters male reproductive efficiency in two ways [18], such as stimulation germ cell number in Sertoli cells (direct way) and elevation of androgen levels by the Leydig cell (indirect way). The word inhibin is derived from inhibit or decline in the levels of the FSH release by negative feedback [19]. Therefore, inhibin is a protein that secreted normally by Sertoli cell in male and by granulosa cell in female. It reduces the FSH release and the LH releasing hormone from hypothalamus [20]. It consists of alpha and beta subunits, as the partial inhibin characterization showed that inhibin forms (a and b) present in alpha subunit, but with different beta one [21]. Some reports have demonstrated the paracrine effect of inhibin on the testicular functionality [22], and the sperm quality [23,24]. A reduction in testicular blood flow in individuals with coronary artery dysfunction was observed when circulating inhibin levels decrease, affecting nitric oxide (NO) and estradiol (E2) levels [25,26]. It has been reported that inhibin could regulates the gonadal development and enhance the animal reproductive capacity [27]. In mammalian studies, the inhibin-related proteins have been shown to function as paracrine signals in the gonads, influencing the generation of testosterone by Leydig cells in the testes and theca-interstitial cells in the ovary [27]. Based on these studies, we hypothesized that immunization against inhibin could affect blood flow, grey image analysis, hormonal aspects, and semen quality in the aged camels. Although the basic function of inhibin, which is to inhibit the FSH, is the same in all species, the precise functions and mechanisms can differ depending on the sex, developmental stage, and the reproductive cycle of the animal. With differences in its function during puberty and in various stages of the estrous cycle, inhibin has a well-studied role in mammals. For instance, the selection of dominant follicles in females is influenced by inhibin levels [28]. Inhibin plays a role in controlling follicular growth and ovulation in birds [29]. The objectives of the study were to determine the effects of active immunization against inhibin on testicular hemodynamics using Doppler technology, and semen picture regarding hormonal profile in male senile camels.

## 2. Materials and Methods

### 2.1. Immunogen Preparation

A synthetic peptide that matches the N-terminal sequence (1–33) of the alpha subunit of pig inhibin was used as the immunogen in this investigation. It was conjugated with rabbit serum albumin, as was previously described in ovine [5]. A volume of Freund’s complete adjuvant (ICN Biomedicals, Inc., Aurora, OH, USA) was added to 100 mg of the synthetic peptide, dissolved in 1 mL of phosphate-buffered saline (PBS) for the purpose of immunization.

### 2.2. Animals, Management, and Immunization

The current study was conducted at King Faisal University, Clinical Studies Department, ALHASA, Saudi Arabia (25°23′ N–49°36′ E) with acceptance and approval from ethical committee deanship of the same university at King Faisal University, Saudi Arabia with an approval number: KFU-2025-ETHICS3331. Ten (n = 10) adult senile male camels (Maghateer; Camelus dromedaries; 700–800 kg; 4 BCS; age is 19–22 ± 0.5 year) were conducted in this study during a period from June 2024 to November 2024. Males were with normal cardiovascular system and normal genital system, including testis and penis. Males were divided into two main groups: First group was served as control (n = 5) and was injected subcutaneous 1 mL saline solution blended in 1 mL Freund’s adjuvant followed by three booster doses of saline solution. Second group was served as immunized group (n = 5) and was injected with 1 mL inhibin vaccine blended in 1 mL Freund’s adjuvant followed by three booster doses of immunization at 4-week intervals. Feeding of animals was consists of 10–12 kg of dry matter, 18–20 kg pasture and free access of water daily. Animals were examined allover twenty weeks at the period from June 2024 to November 2024. The environmental condition such as temperature was controlled as we measured in the fixed time at 6 am before the peak of temperature to avoid any error in the examination.

### 2.3. Inhibin Binding Affinity Assessment

Inhibin binding activity variations in plasma were assessed by evaluating the binding of 125I-labeled inhibin (5000 cpm) as documented earlier [30,31]. Plasma samples mixed at a ratio of 1:10 with the PBS that included 5% BSA. The PBS (100 mL) combined with each aliquot (100 mL) of diluted plasma and incubated for 24 h at 37 °C with 125I-labeled bovine 32-kDa inhibin. Bound tracer was subsequently isolated by the addition of 100 mL PBS with 1%. The mixture centrifuged (3000× *g* for 20 min) after that and radioactivity precipitate was determined and percentage of the binding affinity was determined to the total counting.

### 2.4. Ultrasonography Examination

**Figure 1:**
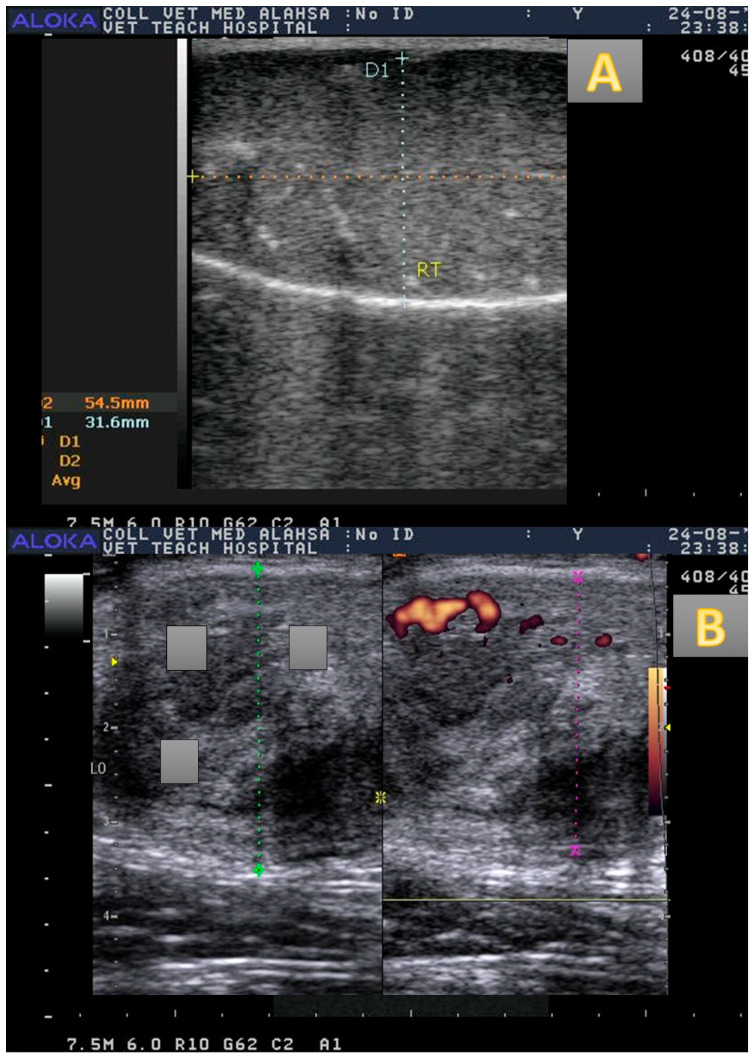
B-mode ultrasound revealed camel testicular length and height to measure the testicular volume (**A**), with a drawing 1 cm^2^ square to estimate testicular echogenicity (**B**) regarding its power Doppler in showing the small vessels.

Camels were presented in the research farm and pushed by an elevator in a setting position to make ultrasound examination and semen collection. Both testicles were examined starting from right side turn to right testicular artery, then the left side was measured with left testicle and left testicular artery. Testicular length (cm), width (cm), and height (cm) were measured (Figure 1A). The caliper was activated in b-mode-frozen image and testicular dimensions were measured. Testicular volume (cm^3^) was also calculated by a known equation of ellipsoid as TV = π abc (4/3), as a, b, and c indicate length, width, and height divided by 2, respectively [32]. No sedation was applied on animals to exclude any alterations on blood supply; all animals were normally heart function to assess any change in blood supply. The scrotum was pushed downward, and the testicle was examined after applying amount of gel.

Grey testicular image analysis of each testicle was estimated using an Aloka ultrasound device (35000SX; Tokyo, Japan). The analysis of grey image depends on testicular tissue echotexture that was measured by drawing square of 1 cm^2^, and then by histogram measurement the echogenicity was determined (TE; NPVs; Figure 1B) as previously calculated [33,34].

**Figure 2:**
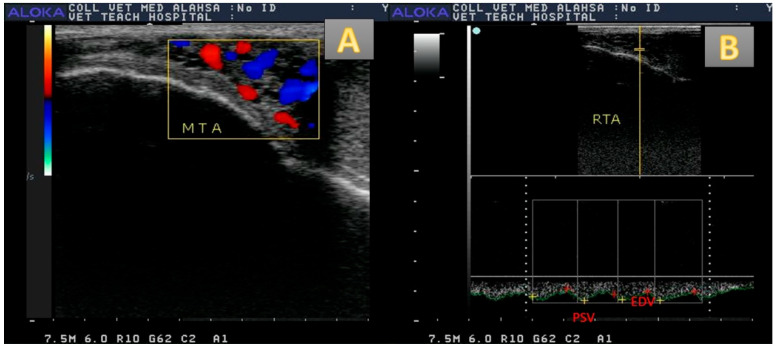
Color Doppler mode ultrasound revealed the pampiniform plexus in camel with coloration (red and blue; (**A**)), and spectral Doppler mode revealed the Doppler measurement in the spectral graph of the known main testicular artery (**B**). PSV = peak velocity (cm), EDV = end velocity (cm).

Color and spectral Doppler modes activated as shown in (Figure 2A,B), with respect to the power Doppler mode (Figure 1B) that represent very small testicular arterioles. The assessment of testicular artery was conducted by activation of a linear probe equipped with 7.5–11 MHz. The location of the artery was determined by the pampiniform plexus as the artery was located at the start of spermatic cord and could be easily detected [35]. The Doppler settings were adjusted to perform optimum measurement as follows: the pulse repetition frequency was 3500 kHz, maximum automated velocity was 35 cm/s, and brightness was 80% with 40° angle of insonation [10,36]. Doppler measurements were summarized as follows: resistance index (RI), pulstatilty index (PI) as those two parameters measured by equation of peak point and end point of velocity (PSV and EDV), in addition to testicular blood flow (TBF; mL/min/100 g) [37].

### 2.5. Blood Collection and Hormone Analysis

Every week before semen collection, blood samples were collected from each camel via its jugular vein at the specific time points (W0, 1, 2, 3, 4, 5, 6, 7, 8, 9, 10, 11, 12, 13, 14, 15, 16, 17, 18, 19, and 20). Each sample was transferred immediately to the laboratory for centrifugation (at 1500× *g*) for 15 min and then the serum samples were kept at −18 °C until further analysis. The FSH was measured in serum samples by radioimmunoassay (RIA) system with 125I-labelled as previously measured in ovine [5,38]. The first antibody used to measure the FSH was anti-bovine FSH, followed by 07RK-550NKK-FSH-I-1 for radioiodination and 07RK-550NKK-oFSH-RP-1, as a reference standard.

The camel Luteinizing hormone (LH) was estimated by an ELISA Kit (SUNLOG; Catalogue Number: SL0024Cm), as previously mentioned with intra-Assay: CV < 12% and inter-Assay: CV < 12% [27,39]. The levels of camel estradiol 17 Beta Dehydrogenase (E2; pg/mL; Catalog No. MBS9381137) and camel testosterone (T; ng/mL; Catalog Number. MBS7606970) were assessed. The laboratory discovered that the intra- and inter-assay coefficients of variation for the focused hormonal and biochemical elements were 4.4% and 5.1% for estradiol, and 5.6% and 8.5% for testosterone, respectively. Before their use with camel serum, the kits were not validated. E2 17-Beta-dehydrogenase, T, sensitivity of 0.1 pg/mL and 0.188 ng/mL for the assay. All measurements were conducted following the manufacturer’s guidelines in the laboratory [40]. The Griess reaction, as measured earlier [41], was utilized to evaluate nitric oxide (NO) samples via Griess reagent.

### 2.6. Semen Collection and Analysis

Two-three semen samples ejaculate were collected once per week after an ultrasound assessment examination. Semen samples were firstly collected by using electroejaculator (EE) into pre-warmed (37 °C) falcon tubes, started from the week of injection (W0) and continued once per week until reaching week 20 (end of the examination). Animal was kept in a setting position after intravenous injection of xylazine 2% (with a dose 0.15 mg/kg) with insertion of electric probe in camel rectum (Minitube, Co, Tiefenbach, made in Germany; 5 cm in diameter and 31 cm in length; with volt 1–10 and 800 mA). Only three runs are performed as the collection procedure was expressed as unsuccessful if more than three runs were utilized [42].

After semen sample collection, the sample was directly transferred to the laboratory and kept in water bath (33 °C), the semen volume (mL) was measured, in addition to PH and semen viscosity (1–5). Semen samples characteristics, such as total motility percent (TM; %), non progressive motility percent (NPM; %), viability percent (V; %), sperm individual motility percent (SIM; %), and sperm cell concentration (SCC; ×10^6^ sperm cell/mL), were measured. All measurements were conducted under a heat stage microscope (Olympus, Tokyo, Japan). Semen was subsequently diluted 1:2 with the same diluent to examine sperm motility (final dilution 1:6, *V*:*V*, the semen to diluent ratio) after total motility was evaluated in an undiluted sample [43]. The components of the diluent were lactose (65 mmol/L), glucose (50 mmol/L), bovine serum albumin (125 mg/100 mL), catalase enzyme (50 IU/mL), and antibiotics (1000 IU/mL penicillin G and 500 µg/mL streptomycin).Then, using a hotplate Olympus CX41RF (Olympus Corporation, Tokyo, Japan), the sperm motility was assessed after 2 μL of diluted semen and was pipetted onto a Leja^®^ Standard Count Slide that was preheated at 37 °C (Leja Products B.V., Nieuw-Vennep, The Netherlands). Sperm motility was evaluated using a hotplate Olympus CX41RF (Olympus Corporation, Tokyo, Japan). About 200 spermatozoa were examined after scanning three random sites on microscope by using CASA system of computer analysis with optimized for camel semen (CASA analysis software system (Intel Macs: CASA 6.7.0, Minitube America; [44]).

Sperm was diluted to perform the CASA analysis to a concentration 30 × 10^6^ sperm cell. The sperm motility parameters were measured as when velocity of spermatozoa less than 5 μm/s is considered immobile, while sperms that showing a velocity between 5–20 μm/s were considered local motile, but those with a velocity more than 20 μm/s were considered motile one classified as locally or non-progressively motile. Spermatozoa with a velocity > 20 μm/s were classified as progressively motile. Semen was subjected to continuous pipetting every five minutes to perform mixture homogeneity as well as reduce the semen sample viscosity [45].

The eosin nigrosine staining technique (composed of 1.67 g eosin, 10 g nigrosine, and 2.9 g sodium citrate dehydrate mixed into 100 mL distilled water) used to evaluate sperm viability percentage [46]. Spermatozoa that stained either partially or fully with pink are considered dead, while live sperm appeared colorless and unstained. All samples obtained with a percentage ranging from (0–100). A minimum of five separate microscopic fields employed to examine triplicate smears of each semen sample.

### 2.7. Statistical Analyses

The SPSS software system version 20 used to conduct analysis and our results proved to be significant at the *p* < 0.05 level, and all values were presented as mean ± SEM. To examine the impact of the immunization as a fixed factor and the time (week) as a repeated factor, means were examined for the difference using repeated measure two-way ANOVA by general linear model of analysis. Along with time points, the Bonferroni post hoc test was used to examine the effects of treatment (two levels; immunized versus control) on changes in testicular blood flow [PI, RI, and TBF], and semen picture [TM, NPM, IM, V, and SCC] in animals as well as on the amounts of circulating hormones [FSH, LH, T, E2, and NO]. Basal levels, often referred to as 0 Week, were calculated by averaging the hormone pretreatment concentrations and the results of Doppler ultrasound tests performed immediately prior to injection.

## 3. Results

### 3.1. Inhibin Antibody Titer and Hormonal Profile

**Figure 3:**
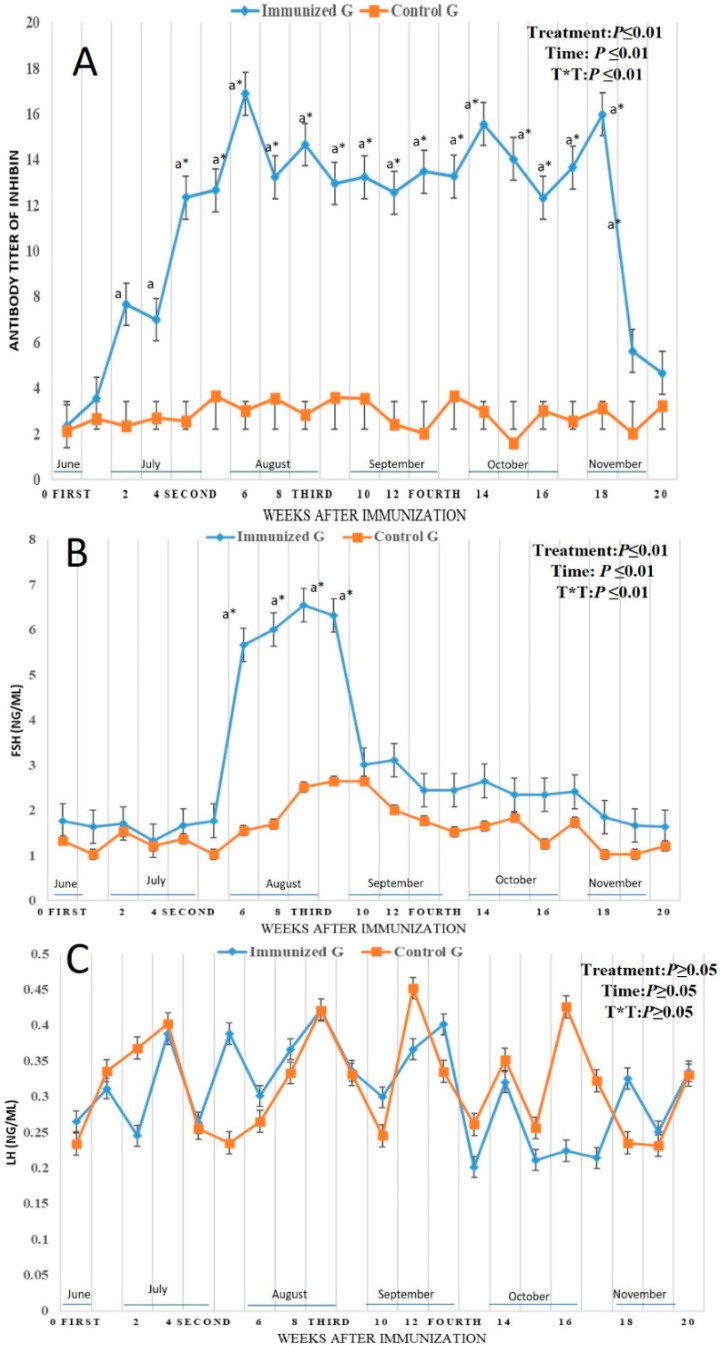
The inhibin antibody titer levels: (**A**) follicle stimulating hormone levels (FSH; ng/mL); (**B**), luteinizing hormone levels (LH; ng/mL); (**C**) in male camel immunized against inhibin a-subunit (immunized G) and control males (Control G) throughout weeks of examinations. Data were obtained as mean ± SEM. The percentage of 125I-labeled bovine 32-kDa inhibin bound at a plasma dilution of 1:10 is used to express inhibin antibody titers. The index ^a^ indicates that there was a significant difference at *p* < 0.01 level in the treated group with time effect compared to week 0, while * indicates that there was a significant difference at *p* < 0.01 level between two groups at the same time point (same week). Immunization times are expressed as first dose at week 0, and other three booster doses at weeks 4 (second), 8 (third), and 12 (fourth).

**Figure 4:**
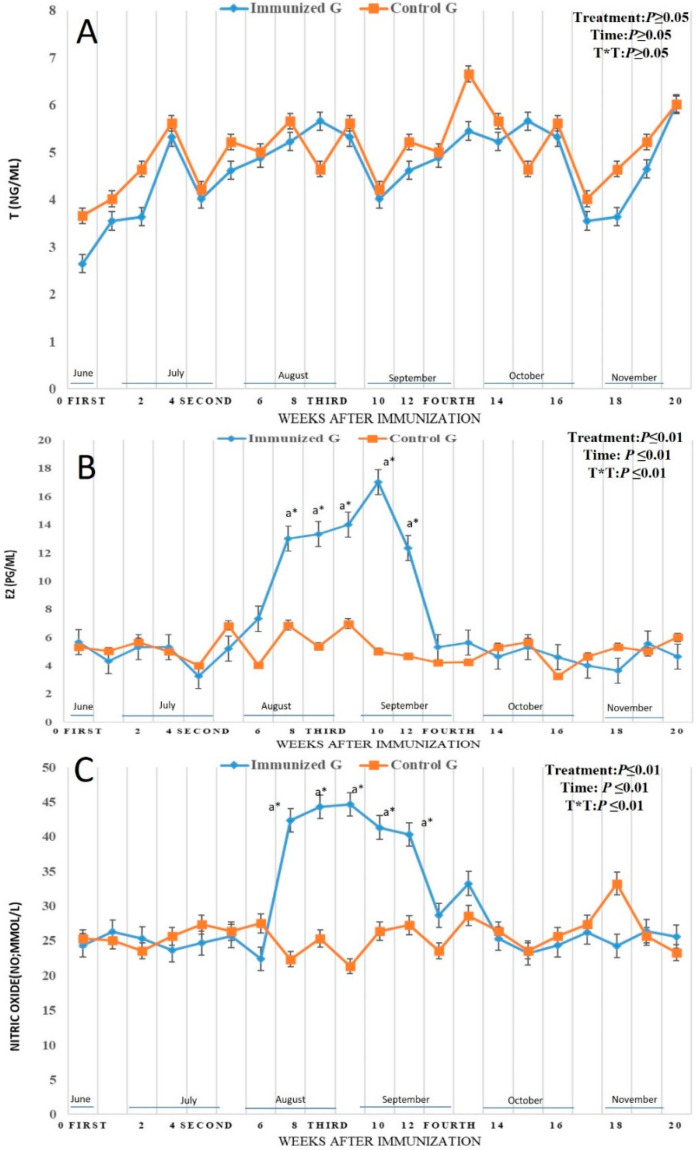
Changes in testosterone levels (T; ng/mL; (**A**)), estradiol 17 α levels (E2; pg/mL; (**B**)), and nitric oxide (NO; µmol/L; (**C**)) in male camel immunized against inhibin a-subunit (immunized G) and control males (Control G) throughout weeks of examinations. Data were obtained as mean ± SEM. The index ^a^ indicates that there was a significant difference at *p* < 0.01 level in the treated group with time effect compared to week 0, while * indicates that there was a significant difference at *p* < 0.01 level between two groups at the same time point (same week). Immunization times are expressed as first dose at week 0, and other three booster doses at weeks 4 (second), 8 (third), and 12 (fourth).

By binding of ^125^ I-labeled bovine, inhibin antibodies were formed in all bull camels that received inhibin vaccine. The effect time was determined, and the titer was increased after first immunization and staying in an elevated pattern until week 18 of the examinations. In the control males, the titer remains in lower levels not changed similar to their levels at preimmunization period. There was treatment time interaction (*p* < 0.01) as well as treatment effect on antibody titer (*p* < 0.01). Antibody titer was elevated from week 2 at July (7.66 ± 0.01) reaching the highest levels in week 6 in August (16.88 ± 0.21), then continued in elevation pattern until week 18 of the examinations in November (Figure 3A). Regarding hormonal levels, follicle stimulating hormones (FSH; ng/mL) was altered in both control and immunized males, and there was a time effect (*p* < 0.01). The FSH was increased in both groups with interaction between treatment and time (*p* < 0.01), but in the immunized group there was a remarkably significant(*p* < 0.01) elevation from week 6 (5.66 ± 0.25 ng/mL) to week 9 (6.23 ± 0.15 ng/mL) in August with the highest level in week 8 (6.54 ± 0.45 ng/mL) (Figure 3B). For luteinizing hormone (LH; ng/mL) and testosterone (T; ng/mL), there was no significant changes occurred with no treatment and time interaction as shown in (Figure 3C) and (Figure 4A) between the two groups. It is surprising that both estradiol 17 α (E2; pg/mL), and nitric oxide (NO; µmol/L) levels were enhanced in the immunized males compared to the control. There was treatment (*p* < 0.01), time (*p* < 0.01), and their interaction (*p* < 0.01) on both parameters. The E2 levels elevated significantly in immunized males started in week 7 (13.02 ± 0.15 pg/mL) in August lasting until week 11 (12.33 ± 0.11 pg/mL) in September (Figure 4B) with the highest elevation in week 10 (17.01 ± 0.11 pg/mL) in September. The same elevation was noticed in the immunized male compared to the control ones as the NO levels was increased significantly (*p* < 0.01) in week 7 (42.32 ± 0.22 µmol/L) in August lasting until week 11 (40.33 ± 0.12 µmol/L) with the highest elevation in week 9 (44.66 ± 0.15 µmol/L) (Figure 4C).

### 3.2. Testicular Vascularization

**Figure 5:**
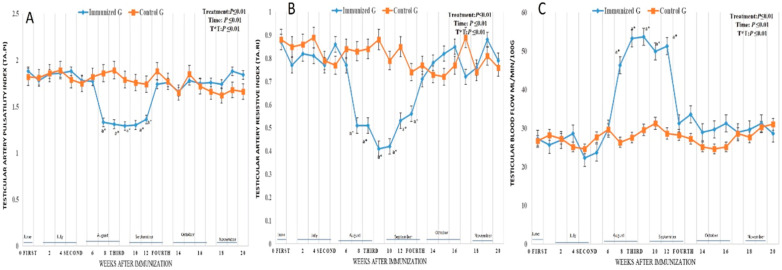
Changes in testicular artery pulsatilty index (TA.PI; (**A**)), testicular artery resistive index (TA.RI; (**B**)), and testicular blood flow volume (TBFV; mL/min/100 g; (**C**)) in male camel immunized against inhibin a-subunit (immunized G) and control males (Control G) throughout weeks of examinations. Data were obtained as mean ± SEM. The index ^a^ indicates that there was a significant difference at *p* < 0.01 level in the treated group with time effect compared to week 0, while * indicates that there was a significant difference at *p* < 0.01 level between two groups at the same time point (same week). Immunization times are expressed as first dose at week 0, and other three booster doses at weeks 4 (second), 8 (third), and 12 (fourth).

Both testicular artery Doppler indices, and blood flow volume were affected by inhibin as there was treatment (*p* < 0.01), time (*p* < 0.01), and their interaction (*p* < 0.01). There was a time effect on testicular artery pulstalilty index and resistive index as TA.PI and TA.RI were significantly declined (*p* < 0.01) from week 7 (1.33 ± 0.01 for PI and 0.51 ± 0.01 for RI) in August until week 11 (1.36 ± 0.01 and 0.53 ± 0.01 for RI) in September with a lowest levels observed in week 9 (1.29 ± 0.01 and 0.41 ± 0.01 for RI) in August (Figure 5A,B). Speaking about testicular blood flow volume, there was a time effect as this parameter showed a significant elevation from week 7 (46.25 ± 0.55 mL/min/100 g) until week 11 (51.22 ± 0.45 mL/min/100 g) with a highest level at week 9 (53.66 ± 0.45 mL/min/100 g) as depicted in (Figure 5C).

### 3.3. Testicular Morphometry and Echogenicity

**Figure 6:**
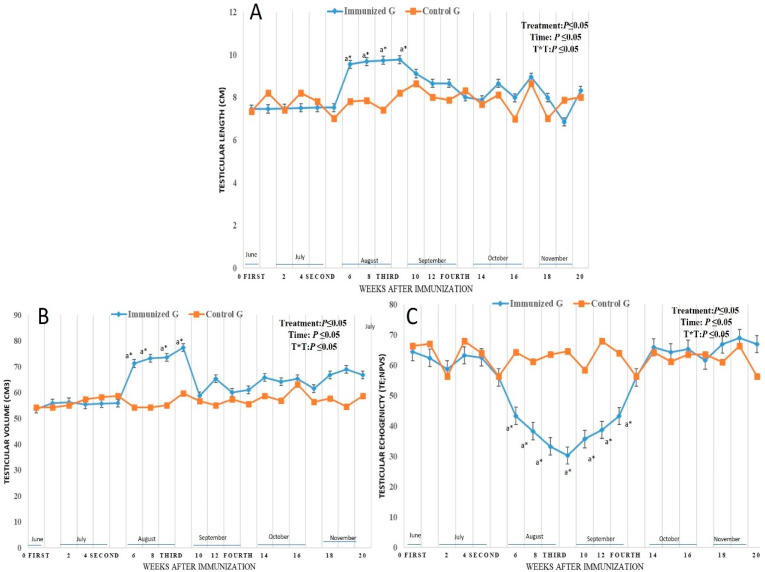
Changes in testicular length (cm; (**A**)), testicular volume (cm^3^; (**B**)), and testicular echogenicity (TE; NPVs; (**C**)) in male camel immunized against inhibin a-subunit (immunized G) and control males (Control G) throughout weeks of examinations. Data were obtained as mean ± SEM. The index ^a^ indicates that there was a significant difference at *p* < 0.05 level in the treated group with time effect compared to week 0, while * indicates that there was a significant difference at *p* < 0.05 between two groups at the same time point (same week). Immunization times are expressed as first dose at week 0, and other three booster doses at weeks 4 (second), 8 (third), and 12 (fourth).

Immunized male camels showed a significant increase (*p* < 0.05) in both testicular length (cm) and testicular volume (cm^3^) form week 6 (9.55 ± 0.11 cm, and 71.22 ± 0.55 cm^3^) to week 9 in August (9.77 ± 0.11 cm, and 77.36 ± 0.45 cm^3^), respectively (Figure 6A,B). There was treatment effect (*p* < 0.05), time effect (*p* < 0.05) and their interaction (*p* < 0.05) between parameter at the time points of examinations. Regarding testicular echogenicity (TE; NPVs), there was a (*p* < 0.05), time effect (*p* < 0.05) and their interactions (*p* < 0.05), as the TE levels was declined significantly from week 6 (43.25 ± 0.11 NPVs) in August to week 12 (43.22 ± 0.55 NPVs) in September with a marked decline in week 9 (30.25 ± 0.51 NPVs) (Figure 6C).

### 3.4. Semen Picture

Immunized male camels showed a significant (*p* < 0.05) higher concentrations of sperm cells, and higher viability percent, from week 9 to week 13 of the immunization compared to the control males that showed no changes in those parameters. The sperm cell concentration (SCC; 10^6^ sperm/mL) was elevated from week 9 (329 ± 5.32 × 10^6^ sperm/mL) reaching the highest value in week 12 (361 ± 10.22 × 10^6^ sperm/mL). Viability (V; %) also elevated from week 9 (78.25 ± 4.22%) to week 13 (80.35 ± 2.35%). As shown in (Table 1), the local non-progressive motility (NPM; %), sperm individual motility (IM; %), and the total motility (TM; %) were not changed significantly in the immunized males compared to the control. However, the volume did not change anymore between groups before and after immunization.

## 4. Discussion

To the best of knowledge, this is the first study conducted on senile male camel related to immunization against inhibin regarding hormonal analysis, testicular artery vascularization, and semen picture. The immunized males showed an elevation in the FSH, E2 and NO levels, especially in August without any substantial changes in the LH, T, and motility parameters.

The immune response noticed after immunization in the antibody titer, as immunized camel formed an antibody against inhibin just after immunization with the first dose and then the levels of antibodies continued in elevation with booster doses of injection. This is similar to lambs and bull calves that both developed higher levels of antibodies after first and second booster doses, respectively [47,48]. Also our data are in line with bucks that developed higher antibodies titer after immunization [5], the immune response in camels, however, could differ from bucks and bulls because of the type of the immunogen used.

The current study showed a trend for seasonal variations in the FSH secretions, this is logical findings as camels are seasonal-breeder animals [49]. Therefore, camel’s active immunization to inhibin can affect markedly the FSH secretions. This agrees with the immunization of shiba bucks against inhibin and the elevation of FSH secretions along time of immunization [30]. The FSH levels significantly elevated from week 6 to week 9 with a marked elevation in week 8 in August. Similarly, bucks were immunized at the age of 3–6 weeks showed increased FSH, starting in week 7 until week 9 [30], which is consistent with the findings for the vaccinated bull [50].

According to earlier research in a variety of species, inhibin’s function has primarily been studied in relation to gonadotropin control and reproductive processes, rather than growth or energy metabolism [38]. Additionally, gonads generate the TGFβ family proteins Inhibin A and B, which inhibit FSH secretion without influencing LH secretion [21]. Both LH and T levels in male camels were unaffected by inhibin vaccination. Other studies have revealed that active inhibin vaccination either had no effect [5] or decreased the LH concentrations [30], despite the fact that Bame et al., [50] reported that bulls inoculated against inhibin had greater levels of testosterone and LH. Ramaswamy et al. [51] reported that inhibin infusion reduced monkeys’ production of FSH but had no effect on LH. Locally generated inhibin may work as a paracrine regulator of testicular function in addition to its endocrine function in controlling FSH secretion. Without changing the levels of FSH, animals treated with intra-testicular inhibin A had fewer spherical spermatids [52].

This current study showed the insignificant change in the T levels, which is similar to previous studies reported that T was not affected by immunization against inhibin as shown in prepubertal bulls [53], and young bucks [54]. There was a study reported the role of inhibin and T secretions [55]. The insignificant alterations in the T levels could be related to an insignificant change in the LH levels. The study’s findings of significant elevation in E2 and NO levels following inhibin immunization were consistent with those found in goats [30], and bulls [50]. According to a study on granulosa cell culture, the inhibin basal local inhibitory effects were prevented by the action of anti-inhibin antibodies [56]. Estrogen is considered as a perfect vasodilator of the reproductive tract with systemic vascularization with the help of nitric oxide (NO). NO synthase plays a role in promoting endothelial nitric oxide generation and reducing the reactive oxygen species [57,58]. Furthermore, in stallions, Bollwein et al., [59] discovered a significant relationship between the testicular artery PI and the total estrogen concentration in the control or hCG-treated groups. In addition, the same pattern was observed in bulls treated with hCG [10].

Some significant alterations were evaluated following immunization in male camels, according to Doppler data. These alterations resulted in a notable increase in the testicular artery blood flow volume and a substantial decrease in the Doppler indices. In accordance with numerous other species that have reported a similar correlation, we thus obtained a negative correlation between the Doppler indices and the vascular perfusion of the primary testicular artery [60,61].

The explanation for why testicular hemodynamics elevates after immunization against inhibin could be related to the elevation of FSH levels that enhances aromatization of androgen conversion into estrogens that elevates both E2 and NO after that [62]. Thus, immunity to inhibin may improve sensitivity to the FSH, which may then encourage cell division and result in larger testes diameter, which is in line with the study’s findings of an increased testicular volume because of immunization. Rams [63], bucks [30], and bulls [50] with active inhibin vaccination had larger scrotal circumferences, which in turn affect testicular dimensions and volume. Bagu et al., [64] recently discovered that giving the FSH to bull calves with ages of between 4 and 8 weeks accelerated the beginning of puberty, improved spermatogenesis, and boosted testicular development.

The analysis of echotexture through computer technology is an impartial approach to assess testicular echogenicity and heterogeneity, serving as an alternative to invasive methods for evaluating male reproductive functions [8,65]. This analytical approach relies on observing variations in pixel gray scale intensity values, and pixel distribution within the frozen ultrasound images of testicular tissue. This cloud be connected to the microstructural characteristics of the testes [66,67]. The testicular tissue echogenicity (TE) decreased in our study, which is consistent with other research that found the TE levels were correlated with maturity. The parameter remained unchanged or even decreased in certain situations [33,68]. The decrease in testicular tissue echotexture in immunized males makes sense because it is linked to senility, just like in senile camels [69].

Many previous studies determined the testicular echotexture in animals and reported the elevation of the parameter during sexual development with enhancement of seminiferous tubules [70].Our results showed that both sperm cell concentrations, and viability percentage were elevated in immunized males at 9–13 weeks from August to September, as previously known that camel is seasonal breeder animal with no elevation of semen picture in summer season [49]. However, with immunization against inhibin we could obtain a clear benefit in semen picture enhancement by sperm cell concentrations. Regarding motility, the percentage of motile and non-motile sperm measured using the CASA analysis software system (Intel Macs: CASA 6.7.0), but we reported that no significant changes occurred in the progressive motile forward sperm. Therefore, according to our results immunization against inhibin could enhance semen picture by improvement of sperm cell concentrations with no effect on fertilization as to perform perfect fertilization, a progressive motile sperm must be present with a power action [71].

The sperm motility is very critical in fertilization and oocyte penetration process, as there was a relation between the sperm motility and fertility [72,73], in addition immunization with some steroid free follicular fluid for bovine could lead to enhancement the production of inhibin antibodies. This led to neutralization of endogenous inhibin and elevated the FSH levels in the immunized animals. Therefore, by time the pregnancy rate was elevated in females that were previously immunized and mated with immunized male [30]. Similar study reported that the contraceptive vaccine could result in reduction of fertility [74]. Another bull study reported that immunization against N-terminal amino acid 30- could alter sperm density without altering sperm motility [63]. The variations in different studies might be because of the type of immunization procedure and the time of vaccination. Fertility testing of these sperm can complement the analysis and determining the extent to which immunization can affect this process will be the topic of future research. More research is required to assess the short- and long-term impacts of active and passive immunization tactics, including various vaccine types and dosages, as well as the immune response over time, to address the lack of influence on motility percentages despite immunization. Understanding the reasons for unexpected effects also requires looking at a wider variety of variables, including host-specific characteristics, vaccination delivery, and individual immune responses.

## 5. Conclusions

We demonstrated the effect of active immunization against inhibin on testicular echotexture, hemodynamics, hormonal aspects, and semen quality for the first time in camel studies. Concurrently, markedly significant increases in the FSH, E2, and NO levels were found. We found that the inhibin immunization affects testicular blood flow (via a reduction in both RI and PI), echogenicity, and sperm cell concentrations, but further investigations are needed to confirm this finding. As this will be looked at in the future, it would be interesting to supplement the findings with the fertility testing of the sperm to ascertain the degree to which immunization could influence this process.

## Figures and Tables

**Table 1:** Semen traits such as [(total motility (TM; %), local non progressive motility (NPM; %), sperm individual motility (IM; %), viability (V; %), and sperm cell concentration (SCC; 10^6^ sperm/mL)] in the immunized group compared to the control males. ^a^ indicates that there was a difference between both immunized and control at (*p* < 0.05). W indicates week.

W	Total Motility (TM; %)		Local Non-Progressive Motility (NPM; %)		Sperm Individual Motility (IM; %)		Sperm Cell Concentration (SCC; 10^6^ Sperm/mL)		Viability (V; %)	
	Immunized	Control	Immunized	Control	Immunized	Control	Immunized	Control	Immunized	Control
0	69.55 ± 1.23	67.9 ± 1.22	11.88 ± 0.77	12.32 ± 1.02	54.52 ± 5.99	56.72 ± 2.61	275 ± 25.32	255 ± 11.25	70.25 ± 5.92	70.88 ± 0.81
1	68.27 ± 1.52	68.75 ± 0.45	11.32 ± 0.66	12.57 ± 1.55	56.16 ± 5.69	56.28 ± 4.32	255 ± 10.25	245 ± 10.52	68.45 ± 7.36	65.65 ± 0.05
2	67.66 ± 1.22	69.61 ± 1.95	11.25 ± 0.05	13.25 ± 1.25	55.75 ± 1.32	54.22 ± 2.99	261 ± 11.25	256 ± 15.25	68.72 ± 10.25	67.25 ± 0.95
3	68.29 ± 5.28	68.75 ± 1.72	12.31 ± 0.21	12.99 ± 1.05	58.32 ± 2.55	56.32 ± 2.69	261 ± 21.02	259 ± 17.32	71.85 ± 8.65	69.25 ± 0.75
4	67.78 ± 5.06	65.77 ± 1.14	12.65 ± 0.25	13.02 ± 0.74	57.78 ± 1.87	55.69 ± 1.32	285 ± 16.88	261 ± 14.99	73.25 ± 7.58	71.32 ± 0.22
5	69.23 ± 2.03	68.15 ± 0.75	12.88 ± 0.77	13.55 ± 0.06	58.47 ± 0.15	56.77 ± 0.32	274 ± 11.26	270 ± 1.32	73.05 ± 1.23	70.88 ± 0.82
6	68.19 ± 5.11	66.45 ± 0.55	13.28 ± 0.77	13.69 ± 0.66	54.77 ± 3.21	54.21 ± 4.33	271 ± 18.65	266 ± 14.25	71.73 ± 2.78	69.65 ± 0.15
7	67.88 ± 5.33	69.28 ± 1.16	12.87 ± 1.22	12.88 ± 0.74	56.92 ± 7.24	56.75 ± 8.25	280 ± 13.52	258 ± 15.01	71.42 ± 2.71	68.25 ± 0.55
8	67.32 ± 2.33	67.25 ± 4.55	13.48 ± 1.75	12.28 ± 1.32	57.21 ± 2.32	54.32 ± 3.66	258 ± 14.66	265 ± 14.65	66.99 ± 1.20	67.58 ± 0.74
9	65.68 ± 5.05	68.15 ± 0.75	12.25 ± 0.15	13.45 ± 1.55	56.66 ± 5.69	56.98 ± 4.32	329 ± 5.32 ^a^	268 ± 17.25	78.25 ± 4.22 ^a^	70.32 ± 0.66
10	65.62 ± 1.88	66.45 ± 0.55	12.65 ± 0.25	13.01 ± 1.02	55.85 ± 1.32	54.32 ± 5.99	336 ± 7.25 ^a^	249 ± 5.62	76.79 ± 1.20 ^a^	68.25 ± 0.54
11	67.25 ± 1.22	66.18 ± 5.76	12.38 ± 0.77	12.65 ± 0.52	58.32 ± 4.55	56.32 ± 5.69	354 ± 14.02 ^a^	251 ± 4.65	80.25 ± 4.22 ^a^	67.25 ± 0.25
12	65.55 ± 5.62	69.43 ± 2.15	13.02 ± 0.54	12.09 ± 0.74	57.28 ± 1.88	55.69 ± 1.32	361 ± 10.22 ^a^	274 ± 15.97	78.25 ± 2.11 ^a^	70.32 ± 0.11
13	66.02 ± 1.55	68.75 ± 1.72	11.25 ± 0.05	13.25 ± 1.25	58.66 ± 0.88	56.77 ± 0.32	354 ± 17.07 ^a^	244 ± 15.21	80.35 ± 2.35 ^a^	71.02 ± 1.62
14	68.33 ± 1.65	65.17 ± 1.04	12.31 ± 0.21	12.99 ± 1.05	57.28 ± 1.88	55.69 ± 1.32	266 ± 23.25	254 ± 15.62	73.65 ± 5.88	71.25 ± 2.85
15	70.32 ± 1.45	68.17 ± 0.25	12.65 ± 0.25	13.02 ± 0.74	58.66 ± 0.88	56.77 ± 0.32	256 ± 24.25	287 ± 2.51	68.65 ± 6.85	67.26 ± 2.55
16	67.52 ± 1.35	66.41 ± 0.15	12.38 ± 0.77	13.75 ± 0.16	54.65 ± 6.32	54.21 ± 4.33	284 ± 15.21	251 ± 5.25	70.22 ± 5.69	68.25 ± 2.35
17	68.33 ± 1.07	69.33 ± 1.46	11.55 ± 0.05	13.25 ± 1.05	51.62 ± 2.36	54.32 ± 0.33	273 ± 15.62	271 ± 6.22	71.65 ± 2.88	70.88 ± 0.82
18	69.36 ± 1.02	67.77 ± 4.45	11.72 ± 0.15	12.78 ± 0.24	59.36 ± 1.66	58.99 ± 0.58	268 ± 21.22	254 ± 8.69	68.02 ± 4.65	69.65 ± 0.85
19	69.87 ± 1.22	69.02 ± 1.44	11.75 ± 0.28	12.55 ± 1.37	57.28 ± 2.33	57.25 ± 2.12	271 ± 12.25	274 ± 18.25	70.55 ± 4.02	70.88 ± 0.03
20	71.33 ± 0.22	70.32 ± 1.44	11.75 ± 0.15	13.55 ± 1.45	52.36 ± 5.02	55.36 ± 1.66	266 ± 15.32	273 ± 2.36	70.33 ± 2.36	66.58 ± 1.22

## Data Availability

The original contributions presented in this study are included in the article. Further inquiries can be directed to the corresponding author.

## References

[1] Liguori I., Russo G., Curcio F., Bulli G., Aran L., Della-Morte D. (2018). Oxidative stressin, aging, and diseases. Clin. Interv. Aging..

[2] Abdelnaby E.A., Fathi M., Salem N.Y., Ramadan E.S., Yehia S.G., Emam I.A., Salama A., Samir H., El-Sherbiny H.R. (2024). Outcomes of dietary alpha-lipoic acid on testicular vascularization, steroid hormones, and seminal quality in aged Baladi bucks. BMC Vet. Res..

[3] Abdelnaby E.A., Alhaider A.K., Emam I.A. (2025). Effect of repeated injection of human chorionic gonadotropin on semen quality, testicular hemodynamics and hormonal profile of senile camel. Vet. Res. Commun..

[4] Kutzler M., Tyson R., Grimes M., Timm K. (2011). Determination of testicular blood flow in camelids using vascular casting and color pulsed-wave Doppler ultrasonography. Veter Med. Int..

[5] Samir H., El Sayed M.A.I., Nagaoka K., Sasaki K., Abo El-Maaty A.M., Karen A., Abou-Ahmed M.M., Watanabe G. (2020). Passive immunization against inhibin increases testicular blood flow in male goats. Theriogenology.

[6] Zhang S., Wang Q., Li Z., Guo Q. (2023). Testicular ischemia associated with IgA vasculitis in a child: A case report and literature review. Front. Pediatr..

[7] Li M., Zhang X., Yan J., Shu H., Li Z., Ye C., Chen L., Feng C., Zheng Y. (2024). Non-invasive ultrasound localization microscopy (ULM) in azoospermia: Connecting testicular microcirculation to spermatogenic functions. Theranostics.

[8] Pozor M., Morrissey H., Albanese V., Khouzam N., Deriberprey A., Macpherson M.L., Kelleman A.A. (2017). Relationship between echotextural and histomorphometric characteristics of stallion testes. Theriogenology.

[9] Pozor M.A., Muehlhaus J., King A., Macpherson M.L., Troedsson M.H., Bailey C.S. (2011). Effect of pentoxifylline treatment on testicular perfusion and semen quality in Miniature horse stallions. Theriogenology.

[10] Abdelnaby E.A. (2022). Testicular haemodynamics, plasma testosterone and oestradiol concentrations, and serum nitric oxide levels in the Egyptian buffalo bull after a single administration of human chorionic gonadotropin. Reprod. Domest. Anim..

[11] Abdelnaby E.A., Emam I.A., Salem N.Y., Ramadan E.S., Khattab M.S., Farghali H.A., Abd El Kader N.A. (2020). Uterine hemodynamic patterns, oxidative stress, and chromoendoscopy in mares with endometritis. Theriogenology.

[12] Venianaki A.P., Barbagianni M.S., Fthenakis G.C., Galatos A.D., Gouletsou P.G. (2024). Ultrasonography of Testicular Maturation and Correlation with Body Growth and Semen Evaluation in Beagle Dog Model. Vet. Sci..

[13] Siqueira L.G., Areas V.S., Ghetti A.M., Fonseca J.F., Palhão M.P., Fernandes C.A.C. (2013). Color Doppler Flow Imaging for the Early Detection of Nonpregnant Cattle at 20 Days After Timed Artificial Insemination. J. Dairy. Sci..

[14] Palhão M.P., Ribeiro A.C., Martins A.B., Guimarães C.R.B., Alvarez R.D., Seber M.F., Fernandes C.A.C., Neves J.P., Viana J.H.M. (2020). Early resynchronization of non-pregnant beef cows based in corpus luteum blood flow evaluation 21 days after Timed-AI. Theriogenology.

[15] Ginther O.J., Utt M.D. (2004). Doppler ultrasound in equine reproduction: Principles, techniques, and potential. J. Equine Vet. Sci..

[16] Ortega-Ferrusola C., Gómez-Arrones V., Martín-Cano F.E., Gil M.C., Peña F.J., Gaitskell-Phillips G., Da Silva-Álvarez E. (2022). Advances in the ultrasound diagnosis in equine reproductive medicine: New approaches. Reprod Domest Anim..

[17] Ribeiro S., Quirino C.Q., Junior A.B., Pacheco A. (2017). Biometry and ultrasound evaluation of testicles and accessory glands in Santa Ines rams Mariana R. Bras. Zootec..

[18] O’Shaughnessy P.J., Monteiro A., Verhoeven G., De Gendt K., Abel M.H. (2010). Effect of FSH on testicular morphology and spermatogenesis in gonadotrophin-deficient hypogonadal mice lacking androgen receptors. Reproduction.

[19] Burger H.G., Cahir N., Robertson D.M., Groome N.P., Dudley E., Green A., Dennerstein L. (1998). Serum inhibins A and B fall differentially as FSH rises in perimenopausal women. Clin. Endocrinol..

[20] Heshmati H.M., Taleb M., Turpin G. (1983). Inhibine: Origine, nature et rôle [Inhibin: Its origin, nature and role]. Presse Med..

[21] Bernard D.J., Chapman S.C., Woodruff T.K. (2001). Mechanisms of inhibin signal transduction. Recent. Prog. Horm. Res..

[22] Stefano Luisi Pasquale Florio Fernando M. (2005). Reis, Felice Petraglia, Inhibins in female and male reproductive physiology: Role in gametogenesis, conception, implantation and early pregnancy. Hum. Reprod. Updat..

[23] Martin T.L., Williams G.L., Lunstra D.D., Ireland J.J. (1991). Immunoneutralization of inhibin modifies hormone secretion and sperm production in bulls. Biol. Reprod..

[24] Voge J.L., Wheaton J.E. (2007). Effects of immunization against two inhibin antigens on daily sperm production and hormone concentrations in ram lambs. J. Anim. Sci..

[25] Reyes J.G., Farias J.G., Henríquez-Olavarrieta S., Madrid E., Parraga M., Zepeda A.B., Moreno R.D. (2012). The hypoxic testicle: Physiology and pathophysiology. Oxid. Med. Cell Longev..

[26] Kocoglu H., Alan C., Ulker Cakır D., Malkoc E., Cosansu K., Kırılmaz B., Ertung Y., Resit Ersay A. (2013). Association between serum inhibin-B levels and coronary artery disease in aging males. Arch. Med. Sci..

[27] Han Y., Jiang T., Shi J., Liu A., Liu L. (2023). Review: Role and regulatory mechanism of inhibin in animal reproductive system. Theriogenology.

[28] de Jong F.H., Grootenhuis A.J., Klaij I.A., Van Beurden W.M. (1990). Inhibin and related proteins: Localization, regulation, and effects. Adv. Exp. Med. Biol..

[29] Lovell T.M., Knight P.G., Groome N.P., Gladwell R.T. (2001). Changes in plasma inhibin A levels during sexual maturation in the female chicken and the effects of active immunization against inhibin alpha-subunit on reproductive hormone profiles and ovarian function. Biol. Reprod..

[30] Medan M.S., Watanabe G., Nagura Y., Fujita M., Taya K. (2006). Effect of active immunization against inhibin on hormonal concentrations and semen characteristics in Shiba bucks. Theriogenology.

[31] Medan M.S., Watanabe G., Nagura Y., Kanazawa H., Fujita M., Taya K. (2004). Passive immunoneutralization of endogenous inhibin increases ovulation rate in miniature Shiba goats. J. Reprod. Dev..

[32] Love C., Garcia M., Riera F., Kenney R. (1991). Evaluation of measures taken by ultrasonography caliper to estimate testicular volume predict daily sperm output in the stallion. J. Reprod. Fertil.

[33] Brito L.F., Barth A.D., Wilde R.E., Kastelic J.P. (2012). Testicular ultrasonogram pixel intensity during sexual development and its relationship with semen quality, sperm production, and quantitative testicular histology in beef bulls. Theriogenology.

[34] Abdelnaby E.A., Emam I.A. (2025). L-Citrulline single intravenous administration promotes testicular blood flow, plasma testosterone, estradiol, circulating citrulline and serum nitric oxide levels in male camels under heat stress at the end of non-breeding season. Reprod. Domest. Anim..

[35] Sary R., Khalil K., Sindi R.A., Mohamed R.H., Hussein H.A., Eid R.A., Samir H., Alkahtani M.M., Swelum A.A., Ahmed A.E. (2022). Characteristics of Ultrasound and Magnetic Resonance Imaging of Normal Testes and Epididymis Besides Angiography of Testicular Artery in Dromedary Camel. Front. Vet. Sci..

[36] Abdelnaby E.A., Alhaider A.K., Ghoneim I.M., Emam I.A. (2024). Vascular Alterations in Uterine and Ovarian Hemodynamics and Hormonal Analysis throughout Pregnancy Loss in Cows under Heat Stress. Vet. Sci..

[37] Salama A., Abdelnaby E.A., Emam I.A., Fathi M. (2022). Single melatonin injection enhances the testicular artery hemodynamic, reproductive hormones, and semen parameters in German shepherd dogs. BMC Vet. Res..

[38] Akhtar M.F., Umar M., Chai W., Li L., Ahmad E., Wang C. (2025). Effect of Inhibin Immunization on Reproductive Hormones and Testicular Morphology of Dezhou Donkeys During the Non-Breeding Season. Animals.

[39] Ali A., Derar D.R., Zeitoun M.M., Al-Sobayil F. (2018). Impotentia generandi in male dromedary camels: FSH, LH and testosterone profiles and their association with clinical findings and semen analysis data. Theriogenology.

[40] Ghoneim I.M., Waheed M.M., El-Bahr S.M., Alhaider A.K., Al-Eknah M.M. (2013). Comparison of some biochemical and hormonal constituents of oversized follicles and preovulatory follicles in camels (*Camelus dromedarius*). Theriogenology.

[41] Fouad K.E., Elzomor S., Farghali H., Emam I., Abdelnaby E.A. (2018). Measurement of normal splenic blood flow indices in donkeys using color Doppler ultrasound. Biosci. Res..

[42] Tingari M.D., El-Manna M.M., Rahim A.T.A., Ahmed A.K., Hamad M.H. (1986). Studies on camel semen, I. Electroejaculation and some aspects of semen characteristics. Anim. Reprod. Sci..

[43] Mansour N. (2023). A novel, patented method for semen collection in dromedary camel (*Camelus dromedarius*). Reprod. Domest. Anim..

[44] Tanga B.M., Qamar A.Y., Raza S., Bang S., Fang X., Yoon K., Cho J. (2021). Semen evaluation: Methodological advancements in sperm quality-specific fertility assessment—A review. Anim. Biosci..

[45] El-Bahrawy K., Rateb S., Khalifa M., Monaco D., Lacalandra G. (2017). Physical and kinematic properties of cryopreserved camelsperm after elimination of semen viscosity by different tech-niques. Anim. Reprod. Sci..

[46] Björndahl L., Söderlund I., Kvist U. (2003). Evaluation of the one-step eosin-nigrosin staining technique for human sperm vitality assessment. Hum. Reprod..

[47] Wheaton J.E., Godfrey R.W., Plasma L.H. (2003). FSH, testosterone, and age at puberty in ram lambs actively immunized against an inhibin alpha-subunit peptide. Theriogenology.

[48] Schanbacher B.D. (1991). Pituitary and testicular responses of beef bulls to active immunization against inhibin alpha. J. Anim. Sci..

[49] Al-Bulushi S., Manjunatha B.M., de Graaf S.P., Rickard J.P. (2019). Reproductive seasonality of male dromedary camels. Anim. Reprod. Sci..

[50] Bame J.H., Dalton J.C., Degelos S.D., Good T.E., Ireland J.L., Jimenez-Krassel F., Sweeney T., Saacke R.G., Ireland J.J. (1999). Effect of long-term immunization against inhibin on sperm output in bulls. Biol. Reprod..

[51] Ramaswamy S., Pohl C.R., McNeilly A.S., Winters S.J., Plant T.M. (1998). The time course of follicle-stimulating hormone suppression by recombinant human inhibin A in the adult male rhesus monkey (*Macaca mulatta*). Endocrinology.

[52] Adeeko A.O., Dunne E., Mather J., Moore A., Morris I.D. (1996). Testicular germ cell populations in the adult rat after continuous in-vivo testicular infusion of inhibin-A and activin-A. Int. J. Androl..

[53] Araki K., Arai K., Watanabe G., Taya K. (2000). Involvement of inhibin in the regulation of follicle-stimulating hormone secretion in the young adult male Shiba goat. J. Androl..

[54] Kaneko H., Yoshida M., Hara Y., Taya K., Araki K., Watanabe G., Sasamoto S., Hasegawa Y. (1993). Involvement of inhibin in the regulation of FSH secretion in prepubertal bulls. J. Endocrinol..

[55] Meachem S.J., Nieschlag E., Simoni M. (2001). Inhibin B in male reproduction: Pathophysiology and clinical relevance. Eur. J. Endocrinol..

[56] Welt C.K., Hall J.E., Adams J.M., Taylor A.E. (2005). Relationship of estradiol and inhibin to the follicle-stimulating hormone variability in hypergonadotropic hypogonadism or premature ovarian failure. J. Clin. Endocrinol. Metab..

[57] Akishita M., Yu J. (2012). Hormonal effects on blood vessels. Hypertens. Res..

[58] Nevzati E., Shafighi M., Bakhtian K.D., Treiber H., Fandino J., Fathi A.R. (2015). Estrogen induces nitric oxide production via nitric oxide synthase activation in endothelial cells. Acta Neurochir. Suppl..

[59] Bollwein H., Schulze J.J., Miyamoto A., Sieme H. (2008). Testicular blood flow and plasma concentrations of testosterone and total estrogen in the stallion after the administration of human chorionic gonadotropin. J. Reprod Dev..

[60] Samir H., El-Sherbiny H.R., Ahmed A.E., Ahmad Sindi R., Al Syaad K.M., Abdelnaby E.A. (2023). Administration of Estradiol Benzoate Enhances Ovarian and Uterine Hemodynamics in Postpartum Dairy Buffaloes. Animals.

[61] Abdelnaby E.A. (2020). Higher doses of Melatonin affect ovarian and middle uterine arteries vascular blood flow and induce oestrus earlier in acyclic ewes. Reprod. Domest. Anim..

[62] Simpson E.R. (2002). Aromatization of androgens in women: Current concepts and findings. Fertil. Steril..

[63] McKeown R.M., O’Callaghan D., Roche J.F., Boland M.P. (1997). Effect of immunization of rams against bovine inhibin alpha 1–26 on semen characteristics, scrotal size, FSH, LH and testosterone concentrations. J. Reprod. Fertil..

[64] Bagu E., Madgwick S., Duggavathi R., Bartlewski P., Barrett D., Huchkowsky S., Cook S., Rawlings N. (2004). Effects of treatment with LH or FSH from 4 to 8 weeks of age on the attainment of puberty in bull calves. Theriogenology.

[65] Arteaga A.A., Barth A.D., Brito L.F. (2005). Relationship between semen quality and pixel-intensity of testicular ultrasonograms after scrotal insulation in beef bulls. Theriogenology.

[66] England G., Bright L., Pritchard B., Bowen I.M., de Souza M.B., Silva L., Moxon R. (2017). Canine reproductive ultrasound examination for predicting future sperm quality. Reprod. Domest. Anim..

[67] Venianaki A.P., Barbagianni M.S., Fthenakis G.C., Galatos A.D., Gouletsou P.G. (2023). Doppler Examination of the Testicular Artery of Beagle-Breed Dogs from Birth to Puberty. Tomography.

[68] Evans A.C., Pierson R.A., Garcia A., McDougall L.M., Hrudka F., Rawlings N.C. (1996). Changes in circulating hormone concentrations, testes histology and testes ultrasonography during sexual maturation in beef bulls. Theriogenology.

[69] Turner R.M. (2019). Declining testicular function in the aging stallion: Management options and future therapies. Anim. Reprod. Sci..

[70] Brito L.F., Silva A.E., Unanian M.M., Dode M.A., Barbosa R.T., Kastelic J.P. (2004). Sexual development in early- and late-maturing Bos indicus and Bos indicus x Bos taurus crossbred bulls in Brazil. Theriogenology.

[71] Scholkamy T.H., El-Badry D.A., Mahmoud K.G. (2016). Developmental competence of Dromedary camel oocytes fertilized in vitro by frozen-thawed ejaculated and epididymal spermatozoa. Iran. J. Vet. Res..

[72] Farrell P.B., Presicce G.A., Brockett C.C., Foote R.H. (1998). Quantification of bull sperm characteristics measured by computer-assisted sperm analysis (CASA) and the relationship to fertility. Theriogenology.

[73] Jasko D.J., Little T.V., Lein D.H., Foote R.H. (1992). Comparison of spermatozoal movement and semen characteristics with fertility in stallions: 64 cases. J. Am. Vet. Med. Assoc..

[74] Gupta S.K., Bansal P. (2009). Vaccines for immunological control of fertility. Reprod. Med. Biol..

